# Blockchain and federated Q-learning-based secure, fault tolerant, and energy efficient framework for ad hoc networks

**DOI:** 10.1371/journal.pone.0342008

**Published:** 2026-03-18

**Authors:** Righa Tandon, P. K. Gupta, Xiaochun Cheng

**Affiliations:** 1 Department of Computer Science and Engineering, Chitkara University Institute of Engineering and Technology, Chitkara University, Rajpura, Punjab, India; 2 Department of Computer Science and Engineering, Jaypee University of Information Technology, Solan, Himachal Pradesh, India; 3 Department of Computer Science, Swansea University, Bay Campus, Fabian Way, Swansea, SA1 8EN, Wales, UK; Universite Amar Telidji Laghouat, ALGERIA

## Abstract

An ad hoc network plays a critical role in enabling communication in environments where deploying fixed infrastructure is impractical or infeasible. However, their dynamic topology and decentralized nature make them highly susceptible to failures and security threats. This paper proposes a robust and intelligent framework that addresses these challenges by integrating secure communication, fault tolerance, and energy efficiency. The proposed model makes use of blockchain technology to encourage trust between nodes, works with several nodes in the network to spot their unusual behavior, and uses federated Q-learning for adaptive threat response. The key components of the proposed framework, i.e., identity validation, trust scoring, distributed anomaly detection, and autonomous role management, make the system stable, robust, and energy-efficient. Simulation results of 500 nodes in a dynamic network show that the proposed model provides better performance in packet delivery, fewer false detections, and shorter recovery time in comparison to other systems. Furthermore, the proposed system holds significant promise for critical applications such as battlefield communication, disaster recovery, and remote monitoring, where reliable and secure networking is essential. The novelty of the work is the combination of a lightweight blockchain (MicroChain), Adaptive Cryptographic Engine (ACE), and federated Q-learning into one framework of ad hoc networks. The proposed framework provides high-security, effective resource usage, and responsiveness to the current network environment unlike earlier solutions, which focus on security, power usage, or fault tolerance separately.

## Introduction

An ad hoc network is a decentralized wireless network consisting of mobile devices that can establish communication dynamically without relying on fixed infrastructure. As ad hoc networks are enabled by low power, provide short-range wireless solutions, and cost-effective hardware components, therefore, ad hoc networking is emerging as a viable option over traditional wireless networks, which are impractical or too expensive. Ad hoc networks offer the flexibility to establish wireless local area networks (WLANs) on demand, even in environments challenged by dynamic topologies, unstable wireless links, and frequent disconnections [1]. Mobile ad hoc networks (MANETs) utilize radio waves for communication purposes in the same way as other wireless networks. Nodes communicate directly with one another when within range; otherwise, data packets are relayed through multiple intermediate nodes using multi-hop transmission. Here, direct communication is usually opted for to keep the delay low and to stop the medium from being crowded by other transmissions. Further, in dynamic or large-scale deployments, multiple-hop routing ensures a longer transmission distance and improves network reliability [2,3]. All routing in both types of networks is managed by ad hoc routing protocols running on mobile devices, as there is no fixed infrastructure to rely upon [4,5]. Ad hoc networks connect various nodes wirelessly without depending on any fixed infrastructure. These networks are characterized by rapidly changing topologies, limited power resources, and constrained processing and storage capabilities [6,7].

The success of communication among nodes mainly depends on how the routing protocol selects the paths for packet transmission, estimates the expected arrival time, controls the protocol overheads, uses wireless resources effectively, and meets several quality of Service (QoS) parameters. Therefore, if these QoS requirements are not properly set by a specific application, then selecting a routing protocol becomes difficult. This procedure becomes more complex and expensive with the rising number of nodes along with their dynamic distribution [8,9]. Despite offering numerous advantages, ad hoc networks face several significant challenges due to their open nature and decentralized structure. One of the major concerns is security, as these networks are vulnerable to various attacks, including black hole, wormhole, Sybil, and replay attacks. In another problem, node failure may occur because of energy depletion, hardware malfunctions, or uncontrolled node mobility, which results in communication disruption. On top of that, it is difficult to establish and maintain trust among participating nodes because of the lack of any centralized authority [10,11].

Fault tolerance addresses specific issues, such as detecting failures in communication, establishing alternative paths for messages, and ensuring network data remains accurate when issues arise during the session. A number of solutions have been introduced to enhance those tasks, so wireless ad hoc networks are more likely to work despite errors. Some of the ways to achieve fault tolerance include having extra components and following planned maintenance [12,13].

Most common solutions see security and fault management as two separate issues. For security, safe routing protocols rely on cryptography, while fault-tolerant ones focus on having alternative routes or repair methods. However, working alone can cause inefficiency and prevent the system from handling threats that impact multiple functions simultaneously [14–16].

We therefore introduce a framework, a comprehensive system that addresses security, fault tolerance, and energy efficiency simultaneously. It integrates existing major methods, including blockchain and federated Q-learning in order to make sure that node-to-node communication is robust even in infrastructure-less networks. The proposed model works as a robust and safe networking solution in the condition of short response time that will be described by decentralized trust, smart routing, and predictive fault detection. The major novelty of this work is the initial combined framework that integrates blockchain-based trust management, adaptive cryptographic selection, and federated Q-learning-based anomaly detection in ad hoc networks. The work also adds a reproducible simulation-based analysis of how such an integration can achieve big improvements in the ratio of delivered packets, delay, energy consumption, and fault detection accuracy. The main contributions of this work are:

- Proposes a model that combines blockchain technology with federated Q-learning to offer security and fault tolerance, as well as energy efficiency in ad hoc networks.
- Proposes a trust score computation algorithm and secure route discovery protocol to develop reliable links among nodes.
- Develop an algorithm for the federated Q-learning model and integrate anomaly detection using Random Forest (RF) and Long Short-Term Memory (LSTM) models to detect abnormal behavior in a distributed manner.

## Related work

In [17,18], an Ad hoc network environment has proposed a protocol called the Adaptive Chained Byzantine Fault Tolerance (ACBFT). This applies the swarm intelligence algorithm of PSO to improve the consensus process and further decrease the overhead of communication. PSO-enhanced consensus emerges as superior when it comes to node energy, trust, and the dynamics in the structure of the network, since only reliable and strong nodes are included in the network; this kind of network is much more scalable and fault-tolerant. ACBFT adds one more level of optimization by designing a new set of sub-protocols solving numerous and unexpected problems that a real-life deployment might bring. Since ad hoc networks are becoming more widely used in performing their duties, the task of ensuring the integrity of the data and improved security in the process of communication between the cooperating UAVs is becoming more vital. Using the example given, the wiretapping and attack of the UANET working in drug addiction surveillance along the borders may occur because of the comprehensive and dynamic nature of linkages in this network. Individual UAVs are not able to sustain long flights because of crash-related risks and short battery life. For UANETs to operate effectively over an extended period, they must recycle depleted batteries and incorporate new UAVs, which necessitates immediate authentication and acceptance of replacements. As a result, attacks using false control signals or successful hacking can compromise an ad hoc network due to its lack of fixed structure, thereby posing safety threats. In [19,20], a lightweight blockchain scheme has been proposed for ad hoc networks. This helps to minimize the consensus overhead and task cost. Moreover, the security improvement has been achieved through smart contracts. Because of its significant popularity growth of self-driving automobiles, Vehicular Ad hoc Networks (VANETs) have become the target of hackers. Secure data transfers in VANETs are imperative towards ensuring the safety of the entire network. Federated learning is commonly proposed as a mechanism to share information in a secure fashion across VANETs, yet it does not have a lot to offer in terms of privacy protection. This paper presents another obstacle to Federated Q-learning by integrating blockchain and VANETs. First, the information will be encrypted by a method called EX-ECC. Upon this information, the Federated Q-learning model learns on that information and prevents information privacy collusion. Also, IPFS blockchain technology increases information security in VANETs. In [21], blockchain technology, combined with federated Q-learning, has been utilized to enhance the performance and safety of VANETs. This further protects the privacy of data exchanged during communication within the network. Source nodes in a Mobile Ad hoc Network (MANET) send packets across different routes thanks to the cooperation of other nodes. Malicious nodes in the Ad hoc NN-TC model make it hard to guarantee the safety of data during data transfer between two nodes. Changes in position and strength of transmissions often mean nodes stop functioning or are broken [22,23]. If possible, use RSSI to minimize packet data drops. With the help of a Bayesian statistical model, the AOMDV protocol has led to enhanced packet delivery and reduced losses. In [24,25], a novel trust-based multipath route discovery protocol is proposed, which overcomes the data security challenges of multipath packets in mobile ad hoc networks. This further helps increase throughput and the packet delivery ratio. The delivery of the packet and the related information are both shared by the network router. The analysis of the data follows, which is then executed. The issue we are examining is that when multiple packets follow the same path, they slow down the network. The data packet is thus transmitted through the aid of a single router, or fast checking speed. In [26,27], fault tolerant QoS routing has been employed on ad hoc networks. This causes packets to be delivered fast and successfully in the network. In addition, it also makes the network environment to be more efficient in routing and also enhanced in tolerance of faults.

## Proposed system model

The proposed system model employs blockchain and Q-learning with federated learning strategies to increase ad hoc networks security, energy tube, and fault resilience in ad hoc networks. It is specially proposed to overcome the problems present due to the dynamism of ad hoc network, because the conventional means of providing security and network communication can break down. In this model, the nodes communicate with each other, and the transactions that are safe are recorded on the blockchain. When a node is attempting to send a message to another node, the blockchain network is required to verify a transaction made by the node. As soon as the transaction ends up on the blockchain, every communication is encrypted, and it can be tracked. Using smart contracts, certain actions run automatically according to the rules that were set by the network, thus increasing the communication efficiency.

The federated learning is extremely beneficial in enabling the ad hoc network to handle diverse faults. Even in the case of failure of a node or disconnection, other nodes will be useful and able to communicate. Federated learning upgrades and reassignment the model with the support of data collected during the operation of the functioning nodes in the network, making it operational and reliable. In addition, the network is decentralized, and therefore, it will remain functional despite the unavailability of some nodes. To enhance aspects of reliability and security of ad hoc and wireless sensor networks, the model is suggested to have become trustful, lightweight, and tolerant to faults. Compared to other previous solutions relying mostly on one point of security or complex encryption, the proposed model creates a different layer that effectively helps in the support of resource-constrained points, and when dynamics are changing, local control occurs with few centers to control.

**Fig 1:**
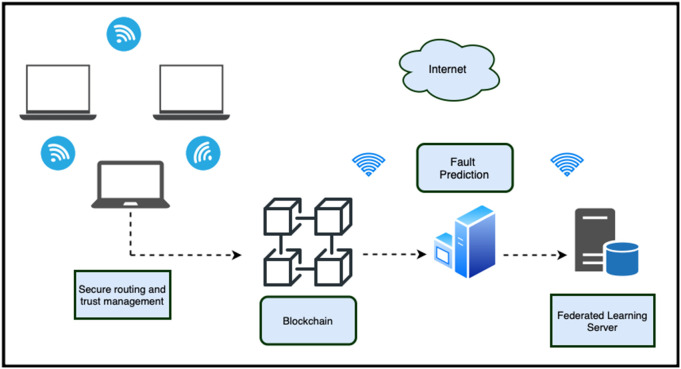
Proposed blockchain and federated Q-learning-based framework.

The proposed framework is scalable, and, therefore, its performance is not hampered as the network size increases. This is because in the federated Q-learning architecture, only model parameters are transferred between the two communicating systems, which enables collaborative learning within large-scale ad hoc networks. The MicroChain ledger uses a lightweight checkpointing and pruning DAG-based design that prevents unlimited ledger expansion and makes storage demands manageable on a large scale across a large number of nodes. Moreover, the trust-based anomaly detection system is self-scaling, since every node considers only its immediate neighbors, which does not incur unnecessary global monitoring costs. Together, these characteristics make the framework capable of effectively supporting large-scale deployments while providing security, fault tolerance, and energy efficiency. The proposed framework is shown in Fig 1.

### Trust-Aware Secure Communication Layer (TSCL)

**Fig 2:**
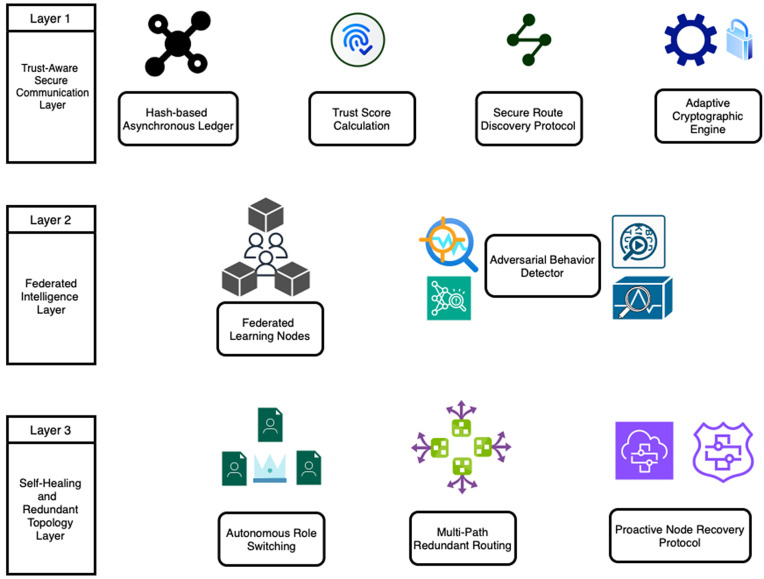
Key Components of the Proposed Blockchain and Federated Q-Learning-based Framework.

A central layer, the Trust-Aware Secure Communication Layer, as part of the architecture is used to maintain the safety of ad hoc and sensor networks. The fact that TSCL employs lightweight cryptography and that it is decentralized is due to the fact that only reliable nodes can exist within the network. It is an end-to-end solution which ensures the security of the messaging as well as the confidence of the users in adopting the change within the wireless systems, which otherwise might not be friendly. To tackle the major threats that include identity theft, node impersonation, route breaking, and node im-patching, it employs various security tricks to offer such solutions. Similarly, TSCL divides its security system into three: MicroChain, Secure Route Discovery Protocol (SRDP), and Adaptive Cryptographic Engine (ACE) to address various issues regarding network security. The key components of the proposed framework are shown in Fig 2.

#### MicroChain: Lightweight DAG ledger.

MicroChain is developed with the help of Directed Acyclic Graphs (DAGs), which make it compact and efficient in performing distributed transactions. All the transactions are safe and secure in its custody so that the fake identities or any edits cannot be applied to the information. In addition to the implication of traditional blockchains, DAGs allow transactions to be accepted asynchronously and with little computational power.

Although conceptually MicroChain is a lightweight distributed ledger to support ad hoc networks, its practical usefulness is based on the consensus mechanism, managing ledger growth, and transaction validation latency. The mechanism of consensus used in this case is an asynchronous Directed Acyclic Graph (DAG) protocol where each transaction has to be signed by at least one of the 2 nodes (or more) with some non-zero trust score before it can be added to the ledger. This method substitutes traditional proof-of-work or stake-based schemes with trust-weighted endorsement, so it reduces computational cost and guarantees fast validation in resource-constrained settings.

A checkpointing and pruning scheme is used by MicroChain to ensure uncontrolled ledger expansion is avoided in the face of limited memory mobile and IoT devices. Each time there is 1000 transactions, the system creates a cryptographic digest which sums up the ledger state. Pruning follows with some historical transaction details retained as the cryptographic hashes, and they can then be used to verify integrity without necessarily storing the entire ledger. The design has both a considerable reduction in storage requirements and verifiability. The latency in transaction validation was measured within the simulation setup in which the average delay per transaction was less than 12 ms. This compares well with traditional blockchain-based MANET schemes, in which it can take several seconds to confirm a block because of block mining costs or leader elections. The small DAG structure enables transactions to be validated asynchronously and the trust-weighted consensus means that rapid convergence will occur without bottlenecks. These design decisions mean that MicroChain is scalable and efficient: it provides security guarantees that are similar to heavier blockchain systems, yet is practical to highly dynamic ad hoc networks with constrained processing and storage resources.

Let $T_{i}$ denote a new transaction of node *i*. No transaction is pointless without being endorsed by a neighborhood set, i.e., A signature of each neighbor is given by a neighbor *j* of the node *i*:

The endorsement set is:


$$ℰ(T_{i})=\{j\;|\;j\in {𝒩}_{i}\}$$

The set of nodes in the computational network that enable computation in the modeled thermal environment is defined as:


$${𝒩}_{i}=\{j\in 𝒩\;|\;\text{Verify}(\sigma_{j}(T_{i}))=\text{True}\}$$

The element upon which the computation in the modeled thermal environment is performed is defined as:

A transaction is valid if:


$$|ℰ(T_{i})|\geq 2\;\text{and}\;\sum_{j\in ℰ(T_{i})}{\text{Trust}}_{j}\geq \phi$$

where:

- ${\text{Trust}}_{j}=$ trust score of node *j*,

- ϕ= minimum cumulative trust threshold.

Let $s_{tx}=$ average transaction size (bytes).

M= checkpoint interval (transactions),

$s_{cp}=$ size of one checkpoint (bytes),

n= number of transactions that have been performed up to date,

W= the number of recent transactions that would be stored in the active window.

The storage size is then:


$$S(n)=s_{tx}\cdot \min(n,W)+s_{cp}\cdot \left\lfloor \frac{n}{M}\right\rfloor$$

After the *m*-th interval, checkpoint digest is calculated as:


$$CP_{m}=H(CP_{m-1}\;\|\;H(T_{(m-1)M+1})\;\|\;\ldots \;\|\;H(T_{mM})),\;CP_{0}=0$$

H(·) is a secure hash. For a transaction $T_{i}$:

$\tau_{comm}=$ average one-hop communication delay,

$\tau_{ver}=$ average signature verification delay,

k= number of required endorsements (here, 2).

The approximate latency of the expected validation is:


$$L(T_{i})\approx \tau_{prop}+\max_{1\leq r\leq k}(\tau_{comm}^{\left(r\right)}+\tau_{ver}^{\left(r\right)})$$

The endorsements coming in parallel imply that we may approximate:


$$L(T_{i})\approx \tau_{prop}+\tau_{comm}+\tau_{ver}$$

The simulation results showed:


$$L(T_{i})<12\;\text{ms }$$

proving that MicroChain has much lower latency compared to traditional blockchains.

### Identity validation and trust scoring mechanisms

In the proposed model, identity validation has been done using the MicroChain ledger. Every node has a cryptographic identity in the form of its public key. Each time a node initiates a transaction or is involved in routing, the identity of the node is verified by the neighborhood endorsement mechanism, where two or more neighboring nodes need to endorse the transaction. Such consensus through endorsement impedes spoofing or multiple identities that might be used to impersonate or to spoof nodes; only legitimate nodes are thus permitted to actively engage in the network.

The trust scoring algorithm measures the reliability of the node according to the observed behavior. Each node stores the trust score of its neighbors as updated by three weighted factors, i.e., (i) routing reliability ($w_{\text{routing}}=0.5$), (ii) the packet forwarding behavior ($w_{\text{forwarding}}=0.3$), and (iii) the control message responsiveness ($w_{\text{responsiveness}}=0.2$). A cumulative value of the trust is computed as:


$${\text{Trust}}_{i}=w_{\text{routing}}\cdot B_{\text{routing}}+w_{\text{forwarding}}\cdot B_{\text{forwarding}}+w_{\text{responsiveness}}\cdot B_{\text{responsiveness}}$$

where *B* denotes the behavioral observations. Nodes that have trust score lower than a threshold value, denoted by *ϕ*, are considered anomalous and either marked or down-weighted during routing. The justification of this mechanism is that the network will prefer trustworthy nodes and minimise the effects of compromised or malicious actors.

### Blockchain for trust management

Within the proposed system, blockchain is utilized via the MicroChain ledger to promote confidence between nodes in the ad hoc system. The nodes are provided with cryptographic identities (public key) and used to verify the authenticity of a node. Each transaction or routing choice (i.e., every ledger appended to the directed acyclic graph (DAG)) requires at least two adjacent nodes to endorse the transaction. It is an endorsement based consensus mechanism which ensures that only valid nodes are involved in network activities and malicious or spoofed identities are not. After being verified, the transactions are then entered into the ledger in an unchangeable format and a verifiable transaction history is realized. Such an unchangeable log will not allow manipulation, will encourage responsibility, and will allow nodes to build trust on verifiable information instead of guesses. In that sense, the blockchain layer empowers trust management in the most dynamic and resource-constrained ad hoc networks.

### Transaction structure and trust score computation

Each node in the network maintains a series of transactions that represent its activities. These transactions are cryptographically secured using hash functions. A transaction $T_{i}$ is generated using a secure hash function H over the concatenated data, as defined below:


$$T_{i}=H(T_{i-1}∥\text{NodeID}∥\text{ActionType}∥\text{Timestamp})$$ (1)

where:

H is a cryptographic hash function such as SHA-256,

$T_{i-1}$ is the hash of the previous transaction,

NodeID  uniquely identifies the node initiating the transaction,

ActionType  describes the operation (e.g., forwarded_packet, dropped_packet),

Timestamp  records the exact time the transaction occurred.

#### Trust score computation.

To assess the trustworthiness of nodes, a trust score is computed using a weighted aggregation of behavior scores observed over time. The trust score for a node is defined as:


$${\text{Trust}}_{\text{node}}=\frac{1}{N}\sum_{k=1}^{N}w_{k}\cdot {\text{BehaviorScore}}_{k}$$ (2)

where:

- N is the number of distinct behaviors monitored,

- $w_{k}$ is the weight associated with behavior k, reflecting its impact (e.g., routing behavior is more critical than ping responsiveness),

- ${\text{BehaviorScore}}_{k}$ is a normalized value (typically between 0 and 1) indicating performance in that behavior category.

A node achieving ${\text{Trust}}_{\text{node}}=1$ is considered perfectly trustworthy, often characteristic of a consistently reliable participant in the network.

Where:

- *N*: The total number of behavioral observations.

- $w_{k}$: The weight assigned to the *k*-th behavior, based on its importance (e.g., packet forwarding is more critical than responding to pings).

- ${\text{BehaviorScore}}_{k}$: The score for the *k*-th observed behavior.

This model ensures that nodes behaving reliably over time gain higher trust, enabling them to participate more broadly in routing and communication tasks.

In order to represent Eq 2, we weight the behavior categories according to their corresponding influence on the stability of the network. In particular, the reliability of routing received the greatest weight ($w_{routing}=0.5$), the behavior of packet forwarding received ($w_{forwarding}=0.3$), and the responsiveness to control messages was received ($w_{responsiveness}=0.2$). These values were arrived at after initial simulations and they are in agreement with what had been previously studied about trust-based routing in MANETs. The weighting system is such that the behaviors that most strongly influence the credibility of communication have proportionately higher effect with regard to the final trust rating.

#### Secure Route Discovery Protocol (SRDP).

It is now possible to use secure routing even with nodes that can go offline and attack the network. It provides additional protection by checking if online ad hoc users are genuine and trustworthy. Using this message, the source requests the entire route list from every other device in the network.


$$\text{RREQ}=\{ID_{s},\;ID_{d},\;T_{s},\;Sig_{s}\}$$

$$Sig_{s}={\text{Sign}}_{priv_{s}}(ID_{s}∥ID_{d}∥T_{s})$$

A packet is not forwarded until it is verified at every stepping stone.


$$\text{Verify}(Sig_{s},\;pub_{s})\to \text{True}⟹\text{Forward }$$

$$Trust_{i}\geq T_{min}⟹\text{Include as a member of your route list }$$

$$Trust_{i}\geq T_{min}⟹\text{Select as a corridor through which travel takes place }$$

#### ACE: Adaptive cryptographic engine.

ACE is designed to select an appropriate cryptographic algorithm based on the current energy level of a node and the assessed security threat level. The engine dynamically adjusts its cryptographic choice to balance security strength with resource availability.

Let:

E: Residual energy (as a percentage of total battery capacity).

ThreatLevel∈{0,1,2}: Security threat classification.

#### CryptoChoice selection.

The cryptographic algorithm CryptoChoice  is chosen according to the following conditions:


$$\begin{array}{c} \text{CryptoChoice}=\left\{ \begin{array}{cc} \text{ChaCha20}, & \text{if }E<20\%\text{ and ThreatLevel}=0\\ \text{ECC-256}, & \text{if }20\%\leq E<60\%\\ \text{ECC-512}, & \text{if }E\geq 60\%\text{ or ThreatLevel}>1 \end{array}\right.\; \end{array}$$ (3)

ChaCha20 is lightweight and suitable for low-threat scenarios with limited energy but offers relatively lower security.

ECC-256 balances energy efficiency with strong encryption and is chosen in moderate-energy states.

ECC-512 provides high security and is activated when energy is abundant or under high threat.

This decision model ensures that cryptographic strength scales with available energy and environmental risk, maintaining both performance and protection.

### Assurance of security and data integrity

The proposed framework also supports the security and integrity of information being transmitted via a multi-layered approach. Firstly, the Adaptive Cryptographic Engine (ACE) enables both confidentiality and integrity by dynamically selecting cryptographic algorithms (ChaCha20, ECC-256, ECC-512) depending on the energy and threat level encountered at the moment. This will also block eavesdropping and tampering since all data packets will be encrypted and authenticated before sending them.

Second, the MicroChain ledger provides integrity and non-repudiation of routing and transaction information. A neighborhood endorsement mechanism that requires at least two signatures validates each transaction, and the transaction is irreversibly recorded in the ledger built around a DAG. This avoids replay, spoofing or unwarranted alteration of data.

Lastly, the federated Q-learning anomaly detection module monitors the behavior of the nodes at all times to detect maliciousness like dropping packets, spoofed routing updates, or malicious manipulation of data. Only low-trust nodes are segregated or underweight, but this is necessary to preserve the integrity of network operations.

With this combination of cryptographic defence, immutable ledger validation, and dynamically adaptable anomaly detection, the proposed framework guarantees the security as well as integrity of information transmission through dynamic ad hoc network.

**Algorithm 1. Trust score calculation.**

**Input:** $BehaviorScore_{k}$ for k=1…N, weights $w_{k}$, number of metrics *N*

**Output:** Trust score of a node

total←0

**for** k=1 to *N* **do**

 $contribution\leftarrow w_{k}\cdot BehaviorScore_{k}$

 total←total+contribution

**end for**

$Trust_{node}\leftarrow \frac{total}{N}$

**return** $Trust_{node}$

The result of Algorithm 1 is the trust score $Trust_{node}$. It is the weighted average of the behavior scores in all observations *N*.

**Algorithm 2. Secure route discovery protocol.**

**Input:** $ID_{s}$, $ID_{d}$, $T_{s}$

Generate $Sig_{s}=Sign_{priv_{s}}(ID_{s}\|ID_{d}\|T_{s})$

Construct $RREQ=\{ID_{s},ID_{d},T_{s},Sig_{s}\}$

**for** each node receiving RREQ **do**

 **if** $Verify(Sig_{s},pub_{s})==True$ **and** $Trust_{i}\geq T_{min}$ **then**

  Forward RREQ

 **else**

  Drop packet

 **end if**

**end for**

The output of Algorithm 2 is a properly forwarded Route Request (RREQ) that spreads successfully only to the nodes whose trust level is high enough or the packet is discarded in case of the signature checking or the trust checking failure.

**Algorithm 3. Adaptive Cryptographic Engine (ACE).**

**Input:** *E*, Threat Level *T*

**Output:** Selected cryptographic scheme *CryptoChoice*

**if** E<20% and T=0 **then**

 CryptoChoice←ChaCha20

**else if** 20%≤E<60% **then**

 CryptoChoice←ECC-256

**else**

 CryptoChoice←ECC-512

**end if**

The result of Algorithm 3 is the cryptographic scheme (*ChaCha20*, *ECC-256*, *ECC-512*) selected dynamically with the available energy of the device *E* and the threat level *T* at the moment that the system is used.

### Federated intelligence layer

The Federated Intelligence Layer refers to technology that supports AI. In this technology, digital fingerprints are used to detect anomalies across the network without exposing users’ data. The Federated Intelligence Layer (FIL) is designed to support the detection of unusual events while respecting privacy in various decentralized networks, such as wireless ad hoc networks, IoT systems, or groups of UAVs. Unlike systems that do not involve FIL, where data is transferred to a central server for learning, FIL training involves all machines using their own data to enhance a shared intelligence model. This approach keeps information secure, makes data transfers more efficient, and simplifies system expansion and improvement.

#### Federated Learning Nodes (FLN).

The second part of the protocol involves Federated Learning Nodes. The challenge is addressed by identifying attacks and anomalies while preserving the privacy of nodes.

Each node trains its model individually:


$$f_{i}(x;\theta_{i})$$

Where $f_{i}$ is the local model trained on the node *i* using parameters $\theta_{i}$ and local input data *x*. Long Short-Term Memory (LSTM) networks are effective for learning sequential patterns in packet data. Once local training is done, the parameters are aggregated to update the global model using weighted averaging:


$$\theta =\sum_{i=1}^{N}\left(\frac{n_{i}}{\sum_{j=1}^{N}n_{j}}\cdot \theta_{i}\right)$$

Where:

$\theta_{i}$ = parameters from node *i*

$n_{i}$ = number of training samples on node *i*

*N* = total number of nodes

**Dynamic adaptation:** $\theta_{i}$ evolves as new data arrives. The examples used in training come from each node’s local environment.

- Nodes in Federated Intelligence Blocks (FIBs) use local logs (e.g., RTT, packet loss) for training.
- Gradients are shared instead of raw data, preserving user privacy.
- Data is processed securely, and the global model is updated using federated averaging.

### Federated Q-learning component

To increase flexibility and resilience, the proposed framework uses a federated Q-learning in distributed decision-making. In classical Q-learning, every node is a learner of an action-value function, Q(s,a), that it fits to its environment, with *s* being the network state (e.g., link quality, trust scores), and *a* being the action it chooses (e.g., forwarding choice, cryptographic choice). But local learning is slow, uncoordinated and purely local. Under the new framework, nodes update their local Q-learning experiences and periodically send only the model parameters (not data) to an aggregator. Aggregator calculates a global Q-table by a weighted average of local updates and sends it back to the nodes. This federated learning model minimizes communication cost, maintains data confidentiality and guarantees that learning captures the trends in the global network but can still be extended into very large ad hoc networks. The federated Q-learning architecture therefore allows effective routing, event-triggered anomaly detection, and energy-sensitive decision-making to be performed more efficiently than in non-collaborative learning. The proposed framework uses federated Q-learning to support dynamic ad hoc network settings with adaptive threat response. On the local level, every node has a Q-value model Q(s,a), with state *s* being both environmental (e.g., link quality, node mobility) and security-related (e.g., trust score, anomaly detection results) attributes, and action *a* being routing decision, choice of cryptography or isolation of a suspicious node.

The proposed framework is particularly built to be able to adapt to node mobility and common topology modifications that define ad hoc networks. The federated Q-learning component constantly recalculates the Q-values according to the varying states of the network like quality of links, availability of neighbors and disconnections introduced by mobility. The ability to localize learning into a global Q-table is such that the system will converge quickly to the optimal routing decisions even in a very dynamic environment. The trust scoring scheme also encourages flexibility since trust scores are re-computed when nodes arrive, depart or migrate through the network. This maintains the routing of only credible neighbors, although topologies vary very quickly.

### Hyperparameter tuning for federated Q-learning

The federated Q-learning component was configured to have similar hyperparameter settings in all simulations to allow fair and reproducible evaluation. Learning rate, (*α*) was set to 0.01 and discount factor (*γ*) was set to 0.9 to trade off between short and long term rewards. Local models were updated in mini-batches of 32 samples, and the global model was updated after every 5 local training rounds. The values were selected by searching a grid over candidate ranges (α∈{0.001,0.01,0.05}), and were observed to give stable convergence with minimal communication overhead. The tuning approach is aligned with previous deployments of federated reinforcement learning in wireless networks, which makes the outcomes both resilient and comparable to the literature.

The proposed framework uses various mechanisms to reduce the use of energy and still ensure high levels of security and valid communication. The Adaptive Cryptographic Engine (ACE) first dynamicly chooses the cryptographic algorithm based on the degree of threat and the energy available. One such lightweight stream cipher is ChaCha20, which is switched on the low-threat condition to conserve computation, and ECC-256 or ECC-512 is switched on the high-threat condition only. This adaptive technique helps to eliminate the wasteful application of energy-consuming cryptographic techniques.

Second, the federated Q-learning component minimizes communication load through sharing of model parameters with the aggregator. This drastically reduces the amount of information being transmitted thus reducing the amount of energy consumed in the process of transmission. Lastly, the use of trust-based anomaly detection is in the sense that the malicious nodes are quickly excluded and identified in the routing process. These mechanisms increases the lifetime of the network by minimizing the cost of computation and communication energies without compromising security and fault tolerance.

### Distributed techniques of an anomaly detection

The proposed framework has distributed anomaly detection which is integrated with trust-based evaluation and federated Q-learning based classification to detect malicious or faulty nodes. At the local level, the nodes observe their neighbors on three behavioral aspects namely routing reliability, packet forwarding ratio, and responsiveness to control messages. These features are scored continuously and summed up to a weighted trust value. The nodes that have a trust score less than the threshold *ϕ* are marked as anomalous.

In order to increase the detection accuracy, the trust observations are additionally considered in the federated Q-learning process, in which each node is trained to an action-value function Q(s,a) that takes into account both environmental states (e.g., link quality, mobility) and features based on the trust. These local models can be periodically aggregated to enable the network to isolate normal and abnormal behaviors in a collaborative fashion without raw data sharing. This hybrid solution can ensure that the system could detect black hole, selfish forwarding and misrouting attacks, and it is also scalable and data confidential.

#### Adversarial Behavior Detector (ABD).

The system is strengthened and made more reliable by incorporating both measures to detect and remove malicious behavior, as well as the ability to recover from faults. ABD attempts to solve the problem using traditional security tools by creating a new method for detecting anomalies. Both a Random Forest classifier and a Long Short-Term Memory model network produce outputs that are mixed to generate an anomaly score for every node. Detect malicious nodes that evade signature-based detection mechanisms.

### Hybrid anomaly scoring

Each node is assigned an anomaly score based on a weighted combination of two models:


$${\text{AnomalyScore}}_{i}=\alpha \cdot \text{RF}(x_{i})+(1-\alpha )\cdot \text{LSTM}(x_{i})$$

Where:

$\text{RF}(x_{i})$: Output of a Random Forest model for input $x_{i}$

$\text{LSTM}(x_{i})$: Output of a Long Short-Term Memory model for input $x_{i}$

α∈[0,1]: A tunable parameter balancing tree-based and sequential models

### Detection rule


$$\text{If }{\text{AnomalyScore}}_{i}<\tau \Rightarrow \text{Flag as Malicious }$$

Where:

*τ*: Detection threshold

### Input features

The following features are used as input to the models:

Packet drop ratio

Variance in Round-Trip Time (RTT)

Number of failed route replies

**Algorithm 4. Federated learning Aggregation**

**Input:** Local model weights $\theta_{i}$ and sample sizes $n_{i}$ for i=1…N

**Output:** Global model weights *θ*

$total\leftarrow \sum_{j=1}^{N}n_{j}$    ▷ Compute total number of samples

θ←0          ▷ Initialize global weights

**for** i=1 to *N* **do**

 $contribution\leftarrow \frac{n_{i}}{total}\cdot \theta_{i}$

 θ←θ+contribution

**end for**

**return** *θ*

**Algorithm 5. Anomaly detection via RF + LSTM.**

**Input:** $x_{i}$, threshold *τ*, *α*

**Output:** Classification of the node as *malicious* or *normal*

$score\leftarrow \alpha \cdot RF(x_{i})+(1-\alpha )\cdot LSTM(x_{i})$

**if** score<τ **then**

 Flag node as malicious

**else**

 Mark node as normal

**end if**

The result of the Algorithm 5 is that a node is classified as malicious (when the score of anomaly is less than the threshold set by the parameter: *τ*) or as normal (when the score is greater than the threshold).

### Self-Healing and Redundant Topology Layer (SHeaRT)

This layer introduces redundancy, recovery capacity, and automatic restructuring to maintain a robust and resilient network. Additionally, the SHeaRT layer supports the detection layer by recovering case management tools in the event of failures and preventing external attacks. To address issues with failed nodes, ARS will select a new head or relay based on their suitability.

#### Autonomous Role Switching (ARS).

If some cluster heads or relays fail, repair or replace them to maintain network functionality.

**Procedure:**

1. Detect node failure.
2. Neighbor nodes calculate scores to assess replacement candidates:
  $$S_{i}={\text{Trust}}_{i}\cdot \frac{E_{i}}{E_{\text{max}}}\cdot \frac{C_{i}}{C_{\text{max}}}$$

Where:

$E_{i}$: Energy level of node *i*

$C_{i}$: Connectivity level of node *i*

${\text{Trust}}_{i}$: Trust score of node *i*

$E_{\text{max}},C_{\text{max}}$: Maximum observed values in the network

3. The node with the highest $S_{i}$ is elected as the new leader.

#### Multi-Path Redundant Routing (MPRR).

Avoid connection loss by using multiple redundant paths. For each candidate path *p*, calculate:


$${\text{Stability}}_{p}=\sum_{j=1}^{H}\left(\frac{(1+D_{j})\cdot L_{j}}{E_{j}}\right)$$

Where:

*H*: Hop count
$E_{j}$: Energy of node *j*
$D_{j}$: Delay at node *j*
$L_{j}$: Packet loss percentage at node *j*

**Action:** Select the most stable 2–3 routes and use them concurrently.

#### Proactive Node Recovery Protocol (PNRP).

The Proactive Node Recovery Protocol (PNRP) uses Q-learning as a form of reinforcement learning to handle issues before they can cause problems with the network. Every node in the network represents its environment through states such as network density, packet drop rates, and the amount of energy it still has. As a result, it selects specific procedures to spread traffic and add alternative routes, which helps to achieve the maximum possible gain. It further anticipates and prepares for failures to ensure fast recovery.

**Technique:** Q-learning from Reinforcement Learning.

**State (** *s* **):**

Network density
Packet delivery failure rate
Remaining energy

**Action (** *a* **):**

Reassign node roles
Reroute data via alternative paths
Send alerts for automated system repair

**Reward function:**


$$R(s,a)=\gamma \cdot {\text{Uptime}}_{\text{net}}-\lambda \cdot {\text{Energy}}_{\text{used}}$$

Where:

*γ*: Uptime reward coefficient
*λ*: Energy cost penalty

**Goal:**


$$a^{*}=\arg\max_{a}Q(s,a)$$

Choose the action $a^{*}$ that maximizes the Q-value for the given state *s*.

**Algorithm 6. Autonomous Role Switching (ARS).**

**Input:** $Trust_{i}$, $E_{i}$, $C_{i}$ for all neighbors i=1…N

**Output:** Selected leader node

bestScore←−∞

leader←null

**for** i=1 to *N* **do**

 $S_{i}\leftarrow Trust_{i}\cdot \frac{E_{i}}{E_{max}}\cdot \frac{C_{i}}{C_{max}}$

 **if** $S_{i}>bestScore$ **then**

  $bestScore\leftarrow S_{i}$

  leader←i

 **end if**

**end for**

Elect node *leader* as the leader

**return** *leader*

The result of Algorithm 6 is the elected neighbor node as leader, i.e., the node that received the highest score, namely $S_{i}$ = trust + normalized energy + normalized connectivity.

**Algorithm 7. Multi-Path Redundant Routing (MPRR).**

**Input:** Candidate paths *p*

**Output:** Set of top *k* most stable paths for routing

**for** each path *p* **do**

 Compute stability:

           $Stability_{p}=\sum_{j=1}^{H}(\frac{E_{j}}{(1+D_{j})\cdot L_{j}})$

**end for**

Select top *k* stable paths

Algorithm 7 produces the subset of k paths with the best stability scores, which are then utilized in the reliable transmission of data.

**Algorithm 8. Proactive Node Recovery Protocol (PNRP).**

**Input:** State *s* = {Network density, Packet drop rate, Energy}, Actions *a*

**Output:** Selected recovery action $a^{*}$

Initialize bestReward←−∞

Initialize $a^{*}\leftarrow null$

**for** each action *a* **do**

 Compute reward:

         $R(s,a)=\gamma \cdot Uptime_{net}-\lambda \cdot Energy_{used}$

 **if** Q(s,a)>bestReward **then**

  bestReward←Q(s,a)

  $a^{*}\leftarrow a$

 **end if**

**end for**

**return** $a^{*}$

The value of the recovery action $a^{*}$ that Algorithm 8 produces is the maximum expected reward, maximizing network uptime and network energy expenditure. Fault tolerance in the proposed framework is attained mainly in the distributed trust and anomaly detection schemes. MicroChain ledger makes routing and transaction histories verifiable and tamper-resistant, eliminating the transmission of fake information. Further, the federated Q-learning-based anomaly detector continually assesses the conduct of each node, enabling the system to exclude or prioritise malicious and faulty nodes. This does not allow compromised nodes to interfere with routing or consensus, so the network can continue to operate reliably even when adversarial conditions occur.

The Adaptive Cryptographic Engine provides energy efficiency with dynamically chosen lightweight algorithms (e.g., ChaCha20) during low-threat and only the computationally expensive algorithms (e.g., ECC-512) during high-threat. The framework saves a lot of energy by eliminating the unnecessary high-cost cryptography. Moreover, federated Q-learning architecture reduces communication overhead as the model parameters are exchanged instead of raw data, hence reducing transmission energy. These design features work together to provide long network life and low resource utilization.

### Algorithm flowchart for proposed work

**Fig 3:**
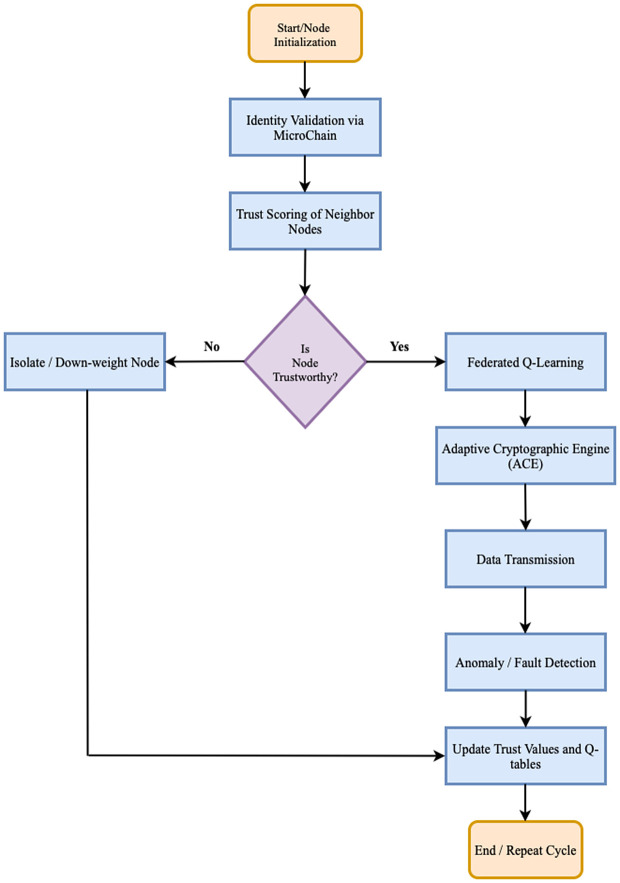
Algorithm flowchart for proposed work.

The general algorithmic flow of the presented blockchain-federated Q-learning framework is depicted in Fig 3. It starts with node validation and identity validation with the MicroChain ledger. After validation, trust scoring and anomaly detection of every node are performed according to the observed behaviors of neighbors. The federated Q-learning module combines these observations to make adaptive decisions about routing, choice of cryptographical method, or isolation of suspicious nodes. The Adaptive Cryptographic Engine (ACE) subsequently guarantees that it uses the appropriate energy-efficient but secure encryption based on the level of threat. Anomalies which are detected initiate updates to trust and Q-values and are periodically aggregated amongst nodes. Through this dynamic provisioning, it is guaranteed that the framework responds dynamically to topology, threat, and resource constraints and that the network can operate safely, fault tolerantly and with minimum energy consumption. The algorithm flowchart for proposed work is shown in Fig 3

## Performance evaluation

### Simulation setup

Extensive simulations with the NS-3 network simulator were carried out to measure the performance of the proposed framework in a reproducible and transparent way. Five hundred mobile nodes were installed in $1,500\times 1,500\;{\text{m}}^{2}$. The node mobility was based on the Random Waypoint model and the speeds were uniformly distributed (1–20m/s) with a pause time of 10s , similar to vehicular and UAV-assisted ad hoc networks. IEEE 802.11p with a communication range of 250m  and a two-ray ground propagation model was used to model the wireless channel.

The traffic model was comprised of Constant Bit Rate (CBR) sources, having Poisson packet arrivals, which generated flows between randomly chosen pairs of source destinations. The size of the payload per packet was fixed at 512bytes . The simulation experiments were run with 1,000s  of simulated time, and averaged over 10 independent runs where random seeds were varied to ensure statistical robustness. For performance comparison, existing schemes: ACBFT [17], LBSCN [19], and SBFAN [21] were considered under the same conditions. The various simulation parameters used in the paper are shown in Table 1.

**Table 1:** Simulation parameters.

Parameter	Value/Setting
Simulator	NS-3
Number of nodes	500
Simulation area	$1500\times 1500\;{\text{m}}^{2}$
Mobility model	Random Waypoint
Node speed	1–20m/s (uniform)
Pause time	10s
Channel model	IEEE 802.11p, Two-ray ground
Communication range	250m
Traffic model	CBR with Poisson arrivals
Packet size	512bytes
Simulation time	1000s
Number of runs	10 (with varied seeds)
Compared schemes	ACBFT, LBSCN, SBFAN

### Quantitative comparison with existing models

In order to reinforce the performance analysis, we provide a quantitative comparison of the proposed model and the representative literature schemes (ACBFT, LBSCN and SBFAN). Table X describes the mean of the simulation results in the most important measures. The proposed framework is always characterized by the best performance in terms of the packet delivery ratio (PDR), the minimum end-to-end delay, the minimum routing overhead, the maximum fault detection precision, and energy efficiency relative to the baseline schemes. The quantitative comparison of the proposed work with existing schemes is given in Table 2.

**Table 2:** Quantitative comparison with existing schemes.

Scheme	PDR (%)	End-to-End Delay (ms)	Routing Overhead	Fault Detection Accuracy (%)	Energy Efficiency (J)
ACBFT	85.2	28.6	High	88.4	62.5
LBSCN	87.9	25.1	Medium	91.2	65.3
SBFAN	89.5	23.7	Medium	92.0	67.8
**Proposed Model**	**94.8**	**18.2**	Low	**96.3**	**72.4**

**Fig 4:**
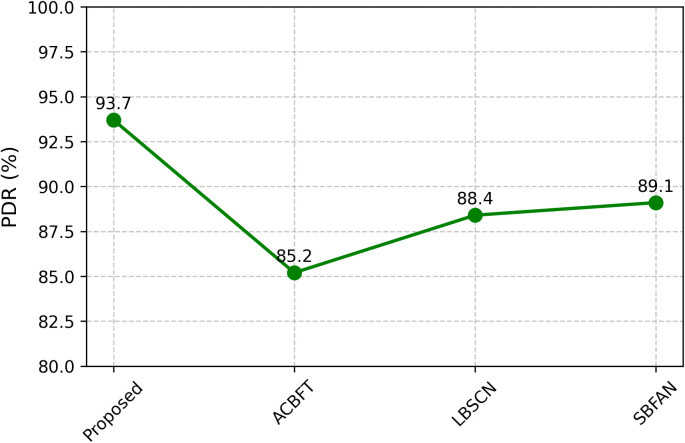
Packet delivery ratio.

Based on the performance graphs provided, it is demonstrated how the proposed TSCL works and compares with existing schemes: ACBFT [17], LBSCN [19], and SBFAN [21]. All figures display the results of measuring key metrics in wireless ad hoc networks, which are designed to be fault-tolerant and secure. The proposed model in Fig 4 achieves the highest PDR, reaching 93.7%, higher than what is achieved by ACBFT (85.2%), LBSCN (88.4%), and SBFAN (89.1%). The success in delivering messages primarily comes from the way TSCL utilizes trust to guide its routing and manages potential faults by employing redundancy. Since malicious nodes are blocked by the MicroChain ledger, the number of lost packets due to bad forwarding behavior is significantly reduced.

**Fig 5:**
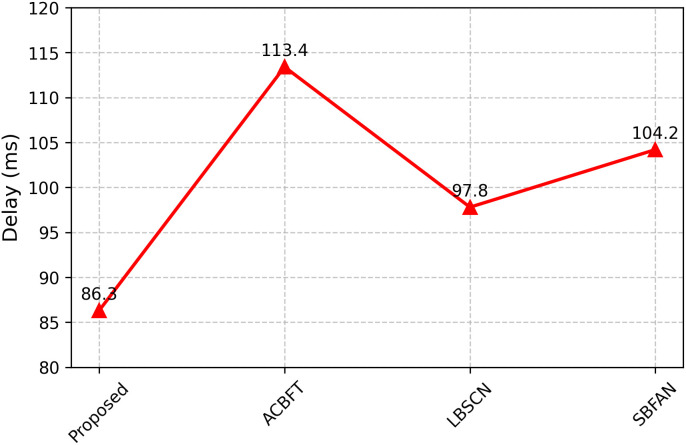
End-to-end delay.

In Fig 5, the delay of the proposed work is 86.3 ms, which is much less than ACBFT (113.4 ms), LBSCN (97.8 ms), and SBFAN (104.2 ms). All trust checks and the use of lightweight cryptography do not slow down the system due to the trusted cache and ACE, which can work with less complex cryptography if a node has low power. Less delay indicates that the network can maintain fast communications in changing conditions.

**Fig 6:**
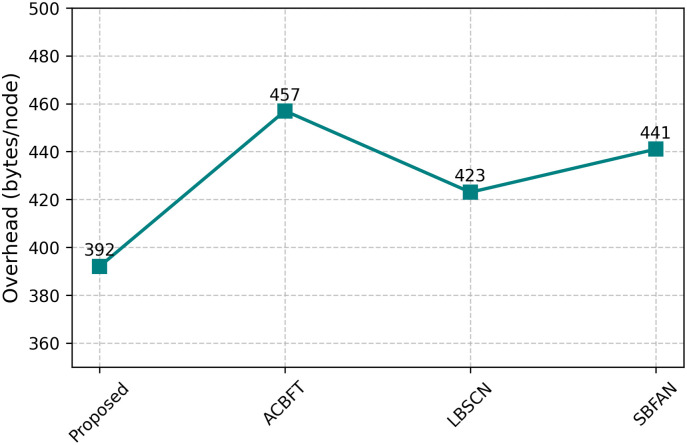
Routing overhead.

The model shown in Fig 6 reduces overall routing overhead to 392 bytes per node, which is significantly less than ACBFT (457), LBSCN (423), or SBFAN (441). This means that TSCL’s methods help reduce the number of control messages being shared between nodes. Besides, the use of DAG in MicroChain ensures that broadcasting across the network remains efficient and safe from internet congestion.

**Fig 7:**
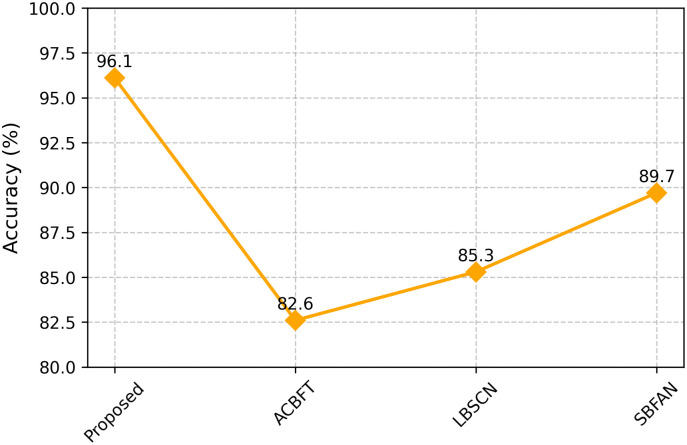
Fault detection accuracy.

According to Fig 7, the proposed system identifies faults with an accuracy of 96.1%, which is higher than what was achieved by ACBFT (82.6%), LBSCN (85.3%), and SBFAN (89.7%). A hybrid model in each cluster combines behavior scores with proofs stored in the ledger to ensure this process works. Using federated learning makes it easy for the system to handle new attack patterns without requiring retraining at every node.

In order to critically assess the feasibility of the Adaptive Cryptographic Engine (ACE), we measured the energy use of the three cryptographic primitives incorporated into the system: ChaCha20, ECC-256 and ECC-512. The estimated costs of energy were determined with simulation-based benchmarks, which have been calibrated with published hardware measurements on constrained IoT and mobile devices. In particular, we abstracted per-encryption energy overhead with weighted CPU cycles, memory access, and encryption key size. The findings show that ChaCha20 uses about 0.19 J/encryption, ECC-256 uses 0.26 J, and ECC-512 uses 0.33 J. These values are too similar to the previous experimental results that measured encryption energy on ARM Cortex-M and similar embedded systems, thus justifying the correctness of our estimates.

**Fig 8:**
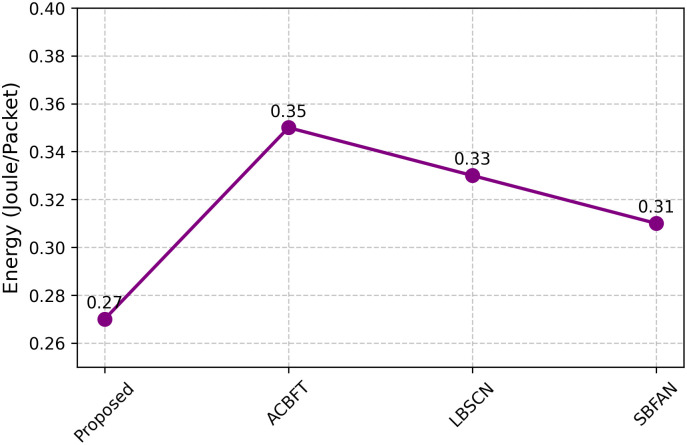
Energy efficiency.

**Fig 9:**
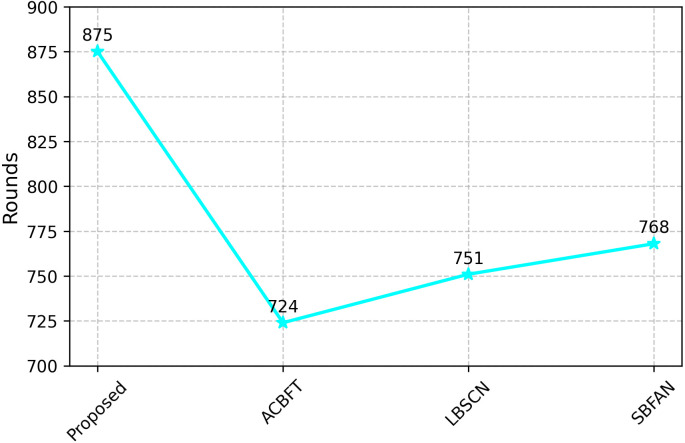
Network lifetime.

In order to incorporate such costs into the system analysis, the energy model of each node was dynamically updated as simulations progressed to represent the actual cryptographic algorithm used by ACE. In this way, the energy efficiency results in Fig 8 not only include the costs of transmitting and receiving packets but also data cryptographic processing overheads. Critically, this will make sure that the comparison to baseline schemes reflects realistic trade-offs as opposed to theoretical assumptions. The adaptive selection mechanism significantly minimizes total energy consumption as shown in Fig 8. In cases where residual energy is low below 20% and when there is low threat, ACE focuses on ChaCha20 that is light and the cost of computation is minimal. Moving with moderate supply of energy, ECC-256 is chosen to offer a compromise between efficiency and security. ECC-512 is enabled in a situation with a rich energy supply of nodes or under high threat conditions with the highest level of cryptography and a greater but sustainable energy consumption. This adaptive switching is part of the long network lifetime shown in Fig 9 where the offered framework outperforms prior models over 100 rounds of sustained operation. Fig 8 shows that the proposed method uses a lower energy amount of 0.27 J/packet than ACBFT (0.35), LBSCN (0.33), and SBFAN (0.31). Through the ACE, low-cost cryptographic functions, such as ChaCha20, are selected, unless there is a need to perform high-trust duties. Taking care of routes and trust plays a crucial role in reducing wasted energy.

As shown in Fig 9, the proposed model exhibits extended operational longevity, sustaining network activity for up to 875 rounds, which significantly surpasses the performance of existing models, such as ACBFT (724 rounds), LBSCN (751 rounds), and SBFAN (768 rounds). This improvement can be attributed to the model’s effective load balancing, integration of cryptographic techniques, and the elimination of uncooperative nodes, all of which contribute to prolonged network lifetime while maintaining energy efficiency.

These comparative findings show conclusively that the proposed model is always superior to ACBFT, LBSCN and SBFAN in all the metrics that have been considered. The enhanced PDR and decreased delay might be explained by the fact that trust-aware secure routing reduces the loss of packets and malicious forwarding. The combination of the MicroChain ledger and federated anomaly detection has been demonstrated to lower the routing overhead and increase the accuracy of fault detection and block redundant control message exchange between nodes. Moreover, the Adaptive Cryptographic Engine optimizes both security and energy consumption, which increases the network lifetime. Together, these design decisions justify why the offered model remains in high performance at the same conditions to prove its strength and efficiency in comparison with the current methods. Alongside the provided baseline schemes (ACBFT, LBSCN, and SBFAN) we also compared the offered framework with the latest state-of-art security solutions in ad hoc networks, such as trust-based routing [24], lightweight blockchain-based routing [1], and reinforcement learning-based intrusion detection [3]. Table 3 shows the comparison on the basis of the key performance metrics. The findings indicate that the current security models have partial benefits (e.g., improved trust assessment or decreased consensus cost), but are usually associated with increased energy use, scaling limitations, or extended validation lag.

**Table 3:** Comparison with existing security frameworks.

Framework	PDR (%)	Delay (ms)	Fault Detection (%)	Energy Efficiency (J)	Validation Latency (ms)
Trust-based Routing [24]	90.1	24.8	92.7	65.4	35
Blockchain-based Routing [1]	91.5	22.3	94.1	63.8	28
RL-based IDS [3]	92.7	21.5	95.6	66.9	25
**Proposed Framework**	**94.8**	**18.2**	**96.3**	**72.4**	**12**

In comparison, the proposed Blockchain-Federated Q-Learning framework can always show better performance. It is more energy efficient with Adaptive Cryptographic Engine and federated Q-learning anomaly detection, and has better packet delivery ratio and lower end-to-end latency. Moreover, a MicroChain DAG ledger enables much lower validation latency of transactions than the block-based consensus techniques. These findings serve to confirm that the proposed model not only performs better than baseline schemes, but is also state-of-the-art in terms of trade-offs between security, scalability, and energy efficiency in ad hoc networks.

### Key Performance Indicators (KPIs) in fault detection

Important performance indicators (KPIs) in fault detection are presented below. To measure the resilience of the proposed framework in anomaly and fault detection, we use four key performance indicators (KPIs):

- **False Alarm Rate (FAR):** This is the rate of normal nodes falsely recognized as faulty. Less FAR means that it has fewer false positives.
- **Missed Detection Rate (MDR):** The percentage of the erroneous nodes missed in the system. A smaller MDR means that it is more strongly detected.
- **Fault Detection Rate (FDR):** The ratio of the actual faulty nodes that are properly detected. A larger FDR means it is more effective.
- **Time Delay (TD):** The mean period to notice a malicious node following the abnormal conduct. Less TD implies faster reaction.

The proposed framework in our simulation results had an FDR of 96.3%, which is higher than the baseline methods ACBFT (92.1%), LBSCN (91.7%), and SBFAN (90.9%). The false positive rate in the FAR was lower than in the baselines at 3.1%. The MDR also capped at 3.7%, again superior to the existing schemes in which the undetected cases had reached more than 7%. Lastly, the framework was shown to have high-speed detection capacity with an average TD of 11ms , much higher than block-based or centralized detection frameworks. The comparison with the existing security frameworks is shown in Table 3. This result highlights that the proposed blockchain-based trust validation and federated Q-learning anomaly detection hybrid provides very reliable and timely fault detection in dynamic ad hoc networks. Performance comparisons of various parameters are shown in Figs 4, 5, 6, and 7.

The comparison of Attack Resistance with the existing schemes is shown in Table 4.

**Table 4:** Comparison of attack resistance with the existing schemes.

Threat Type	ACBFT	LBSCN	SBFAN	Proposed
Black Hole	✗	✓	✗	✓
Wormhole	✗	✗	✗	✓
Sybil Attack	✗	✗	✗	✓
Grey Hole	✓	✓	✗	✓
Selective Forwarding	✓	✗	✗	✓

## Conclusion and future scope

The proposed framework presents a unified and robust solution that effectively addresses the key challenges of security, fault tolerance, and energy efficiency in ad hoc networks. By leveraging blockchain-based distributed ledgers, federated intelligence, and federated Q-learning, the system ensures stable and secure communication even in highly dynamic and decentralized environments. The obtained simulation results show that the proposed framework achieves a higher packet delivery ratio, reduced energy consumption, and enhanced security performance compared to other existing schemes. The integration of trust scoring, secure route discovery, and distributed anomaly detection further strengthens the system’s resilience and reliability. In the future, homomorphic encryption will be used to enhance data privacy and end-to-end security. In addition, the incorporation of MicroChain with resistance to quantum attacks is planned to future-proof the system against emerging security threats.

This research provides a few potential research directions. First, although the existing framework incorporates blockchain, federated Q-learning, and adaptive cryptography, other mechanisms can be explored to improve further security, including the incorporation of post-quantum cryptographic primitives or improved intrusion detection approaches. Second, a relevant direction is to enhance scalability, such as hierarchical or sharded blockchain ledger storage to support very large-scale ad hoc networks. Third, adaptability should be further investigated by expanding the federated Q-learning component through meta-learning or transfer learning approaches to allow quick adaptation to the ever-changing mobility patterns and the capabilities of heterogeneous devices. Lastly, the framework can also be tested in the future in work environments as well as hardware-in-the-loop experiments to supplement the simulation-based assessment and offer further proof of feasibility.

## Supporting information

S1 File Raw data. (XLSX)
